# Cambridge University

**Published:** 1907-09-07

**Authors:** 


### CAMBRIDGE UNIVERSITY.

The degrees of Bachelor of Medicine (M.B.) and Bachelor of Surgery (B.C.) at this University are only conferred after the entire course of study with examinations for both has been carried out. The B.C. is, therefore, in itself a complete and registrable qualification to practise, and is frequently so used. To obtain either of these degrees it is necessary to pass five years in the study of medicine after registration as a student, and to reside for three years at the University. It is not necessary to take any degree or examination in arts, except the Previous examination (or "Little Go"), which is a compulsory preliminary to every degree course, and corresponds to the matriculation of most other Universities. Holders of scholarships are generally required by their college authorities to take an honours course and examination (Tripos) for the B.A. degree, but, apart from this, most medical students at Cambridge do of their own free will take that degree in a Tripos or Pass examination.

Although it is obligatory to reside three years in the University, the student need not do any medical study during that time unless he pleases; the necessary minimum five years of study may be partly or wholly spent in any institution approved by the Board for Medical Studies, and, while there are few who do not spend some part at least of their three years' residence in such study, there are fewer still who do not seek a part of their medical education outside the precincts of the University.

To the Previous examination reference has already been made; this should be passed at the commencement of the first term of residence, unless exemption has already been obtained by some examination recognised for the purpose. The professional examinations are three in number. The first comprises physics, chemistry, and biology; like similar examinations elsewhere, it is divided into two parts, which may be passed separately. Those who have already studied the subjects before coming into residence and have negotiated the Previous examination, will find little difficulty in passing both parts at the end of their first term; for others, a moderate diligence should produce a similar reward at the end of a year. The second absorbs at least two years' work, and frequently more; the standard in anatomy and physiology, which must be passed together, is a very high one indeed, and although the teaching of these subjects in the University is unsurpassed, the percentage of failures in this examination is always large. During the period of preparation for this examination, the medical student who is desirous of an Arts degree must also arrange his studies to that end. He who desires honours in natural science must select either three or four subjects in which to be examined, and for the majority it is most convenient that human anatomy and physiology should be two of these. For the other subjects comparative anatomy, zoology, botany, chemistry, physics, and geology are the favourites with medical students. In the third, or Final examination, there are two parts: one consists of pathology, bacteriology, pharmacology, etc., and may be taken as soon as various courses of lectures and laboratory work in those subjects have been duly attended; the other includes medicine, surgery, and midwifery with gynæcology, and it may not be attempted until the completion of the scheme of medical study, which includes three years' attendance on the practice of a recognised hospital.

In pathology and the other subjects of the first part of the Final examination there are excellent courses of lectures and demonstrations in the University laboratories, and Long Vacation courses in these subjects are especially popular. For the remaining part it is usual to study at one of the large London or provincial medical schools, though there are facilities in the University and at Addenbrooke's Hospital for completing the entire course without leaving Cambridge. This examination is a distinctly searching one, but of an eminently practical kind; the examiners devote themselves to finding out what a man knows of his work, rather than to tripping him on catch points of no intrinsic importance. Many students who present themselves for it have already obtained a registrable qualification from a less exacting corporation and fulfilled a resident hospital appointment; such rarely fail at Cambridge. At the same time an unqualified man has an excellent chance if he has made the most of his opportunities in the wards and out-patient rooms of a large hospital.

When the courses and examinations here sketched have been concluded successfully, the student may take the degree of B.C. at once, but for the M.B. he must prepare and submit a thesis on some subject in medicine, surgery, or midwifery, selected by himself. Although original research is not essential, this thesis must bear evidence that it is founded on the personal observations and contains the personal reflections of the author, otherwise a refusal is certain.

For the degree of M.D. it is necessary to have been M.B. at least three years, and to submit a thesis on some subject in medicine (not in surgery), which is a definite research or contribution to the knowledge of its subject. This must be of a much more elaborate nature than the M.B. thesis. Masters of Arts of four years' standing are allowed to present a thesis for this degree at any time after complying with all the regulations for the degrees of M.B., B.C. A degree of Master in Surgery (M.C.) is also given by the University, as well as diplomas in public health and tropical medicine.

The principal facilities for post-graduate study at Cambridge are rather in the sciences auxiliary to medicine than in any branch of actual therapeutics. In physiology, bacteriology, pathology, etc., there are opportunities of, and equipment for, research unrivalled in the kingdom; studentships and scholarships are awarded for the promotion of this both by the University and by individual colleges. Recently the Lecturer in Pathology, Dr. Strangeways, has collected funds for the scientific investigation of disease; the first subject of this research is osteoarthritis, several sufferers from which are under observation by the lecturer and an endowed assistant investigator. A newly established chair is that of Protozoology; the first professor is Dr. Nuttall, and the pathogenic protozoa are, of course, those to which the greater part of the work done will be devoted.

